# Biosensors and Bioassays Based on Lipases, Principles and Applications, a Review

**DOI:** 10.3390/molecules24030616

**Published:** 2019-02-10

**Authors:** Miroslav Pohanka

**Affiliations:** Faculty of Military Health Sciences, University of Defence, Trebesska 1575, CZ-500 01 Hradec Kralove, Czech Republic; miroslav.pohanka@gmail.com or miroslav.pohanka@unob.cz; Tel.: +420-973253028

**Keywords:** amperometry, bioassay, biosensor, biorecognition, catalysis, enzyme, ester, lipase, nanoparticle, nanostructure, potentiometry, voltammetry

## Abstract

Lipases are enzymes responsible for the conversion of triglycerides and other esterified substrates, they are involved in the basic metabolism of a wide number of organisms, from a simple microorganism and to mammals. They also have broad applicability in many fields from which industrial biotechnology, the production of cleaning agents, and pharmacy are the most important. The use of lipases in analytical chemistry where it can serve as a part of biosensors or bioassays is an application of growing interest and has become another important use. This review is focused on the description of lipases chemistry, their current applications and the methods for their assay measurement. Examples of bioassays and biosensors, including their physical and chemical principles, performance for specific substrates, and discussion of their relevance, are given in this work.

## 1. Introduction

While standard analytical methods and devices for various assays are common in laboratories and specialized workplaces, a single device for reliable handheld assays is not able to cover the whole spectrum of analytes that are measured for diagnostic, environmental, technological and other purposes. For the reason, hand-held devices for the most relevant analytes are demanded. Most biosensors belong to the group of handheld assays and there are also significant off-the-shelf devices conforming to the description of a biosensor.

Biosensors are a combination of two important parts with unique properties [1,2]. The first part is a sensor, also called a physico-chemical transducer, this part is responsible for providing a physically measurable signal. Biological origin is the second compartment of a biosensor and this part is necessary for providing specificity to an analyte. Lipase can be entitled as one of the parts of biological origin.

**Figure 1:**
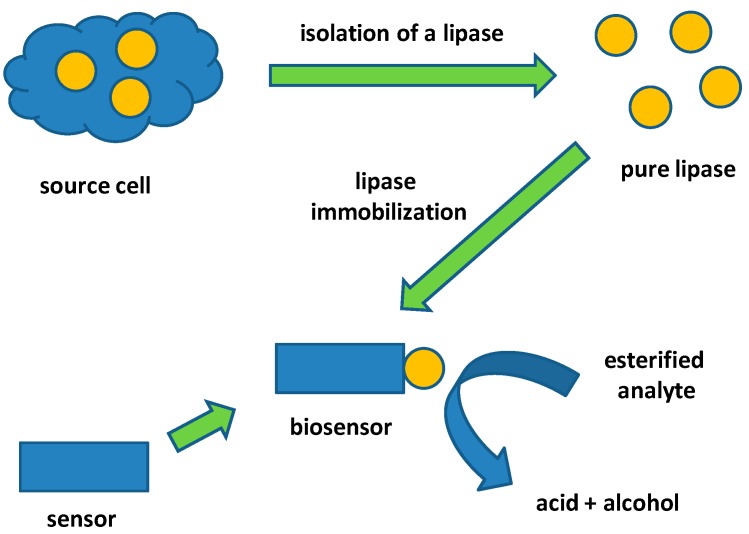
A general idea of a lipase biosensor construction.

Lipases are frequently used in biosensors construction because of their wide substrate specificity and good commercial availability. Various source cells can be used as a source of lipases. Bacterial, fungal and mammal lipases such as the one from the porcine pancreas are available [3,4,5]. Once a lipase becomes a part of the biosensor, it can be used for an assay of either enzymatic substrate or an inhibitor [6,7,8]. A general principle of biosensors with immobilized lipase is depicted in Figure 1.

This review is focused on the survey of actual literature in the field of lipase biosensors, bioassays and similar tools and methods. Discussion of their applicability and suitability in the current analytical praxis is also included in this work. A search in a recent paper on the issue with proper citations is presented here.

## 2. Lipases and Their Applicability

Lipases are a group of enzymes belonging to the lipase-esterase superfamily and having basic structural motives common in this superfamily such as serine in the active site and in the similar way that they are folded after the expression of genetic information [9,10]. Lipases are structurally very close to cholinesterases: acetylcholinesterase and butyrylcholinesterase are both widely applicable for biosensor construction because of their sensitivity to neurotoxic compounds [1,11,12,13]. The example of cholinesterases shows where the development of lipase in bioassays and biosensors can go because they are more advanced in their development due to their use in the assay of military nerve agents and pesticides.

In mammals, pancreatic lipase, lipoprotein lipase and hepatic lipase are distinguished [14]. The most frequently applied lipases are of the proper-named triacylglycerol acyl hydrolases (E.C. 3.1.1.3) and can be easily acquired from the many organisms from which both microorganisms and livestock are most common. While microbial lipases can be produced by standard biotechnological processes. Mammalian lipases can be isolated from livestock organs and agriculture is actually the major source for this type of enzyme. Vertebrate lipases share large sequence identities, ranging from 58% to 97% [15]. The lipase E.C. 3.1.1.3 is nevertheless structurally distinct to lipoprotein lipase EC 3.1.1.34 [15,16].

Triacylglycerol is the main substrate of lipase and glycerol and carboxylic acids are the most important products of the catalyzed reaction. The role of lipase relates to basal metabolic processes and it is involved in retinol biosynthesis, triacylglycerol degradation and lipid metabolism. The common chemical reactions catalyzed by lipase may differ in various organisms. Acetylation [17], the hydrolysis of various carboxylic esters [18,19,20] and transesterification [21,22] are the most typical reactions catalyzed by lipases.

The range of substrates converted by lipases can be learned from the following examples. In the work by Lianghua and coworkers, ibuprofen in R and S racemate reacts with 1-propanol in the presence of lipase from *Penicillium expansum* [23] while (*S*)-ibuprofen n-propylester and (*R*)-ibuprofen are the products. The reaction can be applied for the preparation or manufacturing of an optically pure isomer of ibuprofen. The hydrolysis of 4-nitrophenyl palmitate providing 4-nitrophenol and palmitate was described in the same paper. The high activity of lipase can be found in yeast and it can be used for technological purposes. In a paper devoted to the research on this issue, water hydrolysis of 1,2,3-trihexaicosanoylglycerol to 1,2-dihexaicosanoylglycerol and hexaicosanoate, the hydrolysis of trimyristin to dimyristin and myristate, and the hydrolysis of tripalmitin to dipalmitin and palmitate are described for lipase from yeast *Saccharomyces cerevisiae* [24]. Tributyrin can be designated as a common substrate for lipases of various origin. In a study, tributyrin hydrolysis to dibutyrin and butyrate was mentioned for lipases from various organisms, including bacterium *Bacillus subtilis* and *Chromobacterium viscosum*; funguses *Rhizomucor miehei*, *R. oryzae*, *Fusarium solani*, and mammals wild boar (*Sus scrofa*); and humans *Homo sapiens*, was described [25]. The reaction can repeat up to the full hydrolysis of tributyrin. Tributyrin hydrolysis was described in the work of Fernandez and coworkers for *Micrococcus* sp. as well [26]. The work of Fernadez and coworkers also describes the α/β-naphthyl stearate, β-naphthyl butyrate, and β-naphthyl laureate water hydrolyzes to α/β-naphthol and stearate, β-naphthol and butyrate, and the β-naphthol and laureate water hydrolyzes by *Micrococcus* [26]. An extensive search on substrates for a lipase from beetle *Cephaloeia presignis* was made by Arreguin-Espinosa and coworkers [27]. Water hydrolysis of 4-nitrophenyl laurate to 4-nitrophenol and laurate, 4-nitrophenyl oleate to 4-nitrophenol and oleate, 4-nitrophenol palmitate to 4-nitrophenol and palmitate, 4-nitrophenyl propionate to 4-nitrophenol and propionate, α-naphthyl acetate to α-naphthol and acetate, methyl acetate to methanol and acetate, methyl butyrate to methanol and butyrate, methyl laurate to methanol and laurate, methyl palmitate to methanol and palmitate, methyl propionate to methanol and propionate, and methyl stearate to methanol and stearate was proved. The survey of typical substrates and reaction products related to lipase is given in Table 1.

Some compounds exert affinity toward lipases and they can inhibit them. The inhibitors can have a physiological function in the triglyceride metabolism but beside the natural inhibitors, there are artificial inhibitors serving as, for instance, drugs, natural compounds and pesticides [28,29,30,31]. With regard to biosensors, they can either influence an assay and cause false negative results when an enzyme substrate is analyzed or they can be directly measured when the inhibitor is an analyte and the substrate serves for activity visualization or determination only. The inhibitors of lipases are a wide group of substances from metal elements to organic compounds that can be learned of from cited studies describing the inhibition of a specific lipase by a specific chemical substance. Cadmium (II+) inhibits lipase from *Yarrowia lipolytica* [32], cobalt (II+) inhibits lipase from *Mycobacterium tuberculosis* [33], iron (III+) inhibits lipase from *Pseudomonas fragi* [34], and mercury (II+), nickel (II+), copper (II+) and zinc (II+) inhibits lipase from *Yarrowia lipolytica* [35]. Alginic acid inhibiting lipase from wild boar (*Sus scrofa*) [36], sodium dodecyl sulfate, *n*-dodecyltrimethylammonium bromide, and sorbitan monooleate inhibiting lipase from *Pseudomonas fragi* [34], cetyltrimethylammonium bromide inhibiting lipase from *Bacillus cereus* [37], sodium cholate, sodium lauryl sulfate, *p*-hydroxymeruribenzoate, *N*-bromosuccinimide, acetylacetone, 4-dimethylaminobenzaldehyde from *Rhodotorula mucilaginosa* [38], galacturonic acid and pectin inhibiting lipase from wild boar (*Sus scrofa*) [39], 1-butanol, 1-propanol, 2-propanol, acetone, acetonitrile, benzyl alcohol, iso-amyl alcohol, iso-butanol, phenylmethylsulfonyl fluoride inhibiting lipase from *Geobacillus* sp. [40] can be mentioned as other inhibitors. Some inhibitors can be used as drugs preventing fat metabolism and, thus, work well for weight loss therapy. Orlistat, also known as tetrahydrolipstatin, is such drug. The inhibitory effect of orlistat is well described for lipase from various sources such as the whiteleg shrimp (*Litopenaeus vannamei*) [41], the fungus *Rhizopus oryzae* [42], and, of course, human lipase [43]. The survey of lipase inhibitors is given in Table 2.

In the current economy, it is estimated that the production and sale of lipases is around 10% of all marketed enzymes [44]. The application in the food, beverages and animal feed industry is the most typical for lipases and represents around 55% of marketed lipases. The use in the fabrication of cleaning agents is approximately 25% of the lipase market and the production of biofuels is 10%. Regarding its application in modern biotechnology, microbial lipases prevail as the most marketed type of lipases [44,45,46]. It is estimated that 90% of sold lipases have a microbial origin [44].

Analytical chemistry is not a major issue in why lipases are commercially available. On the other hand, lipases are widely linked to analytes for three reasons. Firstly, the lipase itself is an analyte and the activity of a lipase isoform serves for the control of a biotechnological process, the diagnosis of a disease or the distinguishing of a kind of cell or microorganism. Secondly, lipases are a tool to hydrolyze esters and esters are analytes. The third potential role of lipases in analytical chemistry is in the assay of lipase inhibitors. Just the aforementioned substrates and inhibitors of lipases can be measured by an assay where lipases work as the recognition element. Various methods suitable for the lipase activity measurement are available and they were extensively reviewed by Stoytcheva and coworkers [47]. The relevant methods are introduced in the next chapter.

## 3. Standard Methods for Lipase Activity Assay

Lipase activity assay is performed for many purposes and it is quite standard in microbiology and biochemistry laboratories. Standard microbial tests are performed in order to determine whether the isolated strain has a high, low or no visually detectable activity of lipases and it serves for the closer characterization or selection of bacterial strains that have biotechnological applicability. The tests are typically organized as the visually well-controllable melting of the lipidic cloud in the culturing medium. Tween 20, tributyrin and vegetable oil can be mentioned as relevant compounds added to culturing agars [48].

**Figure 2:**
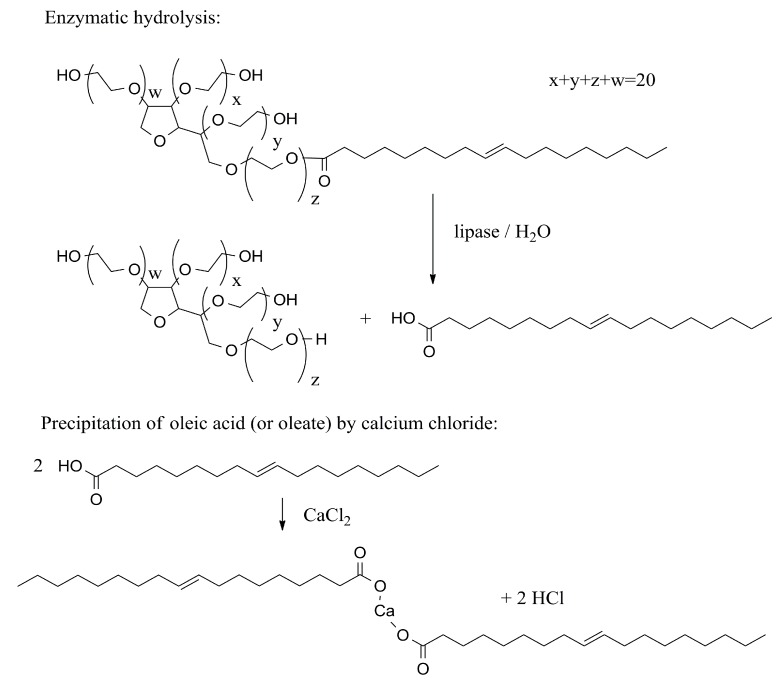
The lipase assay using tween 80 and calcium chloride. The arising calcium oleate can scatter light.

Standard tests in biochemistry are based on changes in the physico-chemical properties of the medium. Typically, the effect of lipase on medium conductivity, optical properties and pH are common. The determination of lipase can be done in the presence of polysorbate and a calcium salt providing the precipitate of calcium and a fatty acid that scatters light. A lipase activity assay where tween 20 (polysorbate 20) or tween 80 (polysorbate 80) and calcium chloride were used are described in the cited papers [49,50]. In the case of tween 80, oleate is formed in the step catalyzed by lipase and then calcium oleate arises by a spontaneous reaction between calcium chloride and oleate and an absorbance around 450 nm can be measured [50]. The principle of lipase activity assay using polysorbate 80 is depicted in Figure 2.

**Figure 3:**
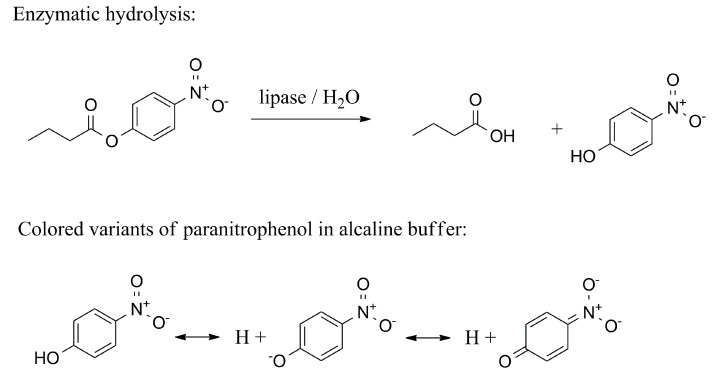
The lipase assay using *p*-nitrophenyl butyrate. The arising *p*-nitrophenol respective *p*-nitrophenolate absorbs light around 410 nm.

Esters of *p*-nitrophenol with organic acids serve as another chromogenic substrate for the determination of lipase activity. This assay can be used for the measurement of lipase E.C. 3.1.1.3 but it is also suitable for the determination of the lipoprotein lipase E.C. 3.1.1.34 activity assay. The principle of the assay is, for instance, well described in the paper by Glogauer and coworkers [51]. In the assay, *p*-nitrophenyl acetate, *p*-nitrophenyl butyrate, *p*-nitrophenyl valerate, *p*-nitrophenyl caproate, *p*-nitrophenyl decanoate, *p*-nitrophenyl dodecanoate, *p*-nitrophenyl myristate and *p*-nitrophenyl palmitate were introduced and tested as substrates for lipase. The assay can be described for *p*-nitrophenyl butyrate as follows. *P*-nitrophenyl butyrate is hydrolyzed by lipase in the water ambient condition, giving rise to *p*-nitrophenol and butyric acid. *P*-nitrophenol actually occurs in the anionic form, *p*-nitrophenolate, giving a yellowish coloration and absorbing light at 410 nm in an alkaline buffer such as Tris-HCl pH 7.5. The principle of the assay is shown in Figure 3.

The titrimetric assay using a triacylglycerol is another reliable method [51,52]. In the assay, glycerol and the appropriate acid are released and the pH of the medium decreases and is titrated by an alkaline solution. The major advantage of the titrimetric assay is the resistance to solution turbidity. Beside these advantages, the assay also has a disadvantage as the strong buffers make it hard to hold a stable pH, making it harder for the assay to be performed. Substances influencing the pH of the solution are another issue. Trioleoylglycerol and tributyrylglycerol can be mentioned as convenient substrates for the titrimetric lipase assay [53].

## 4. Electrochemical Lipase Biosensors and Bioassays

**Figure 4:**
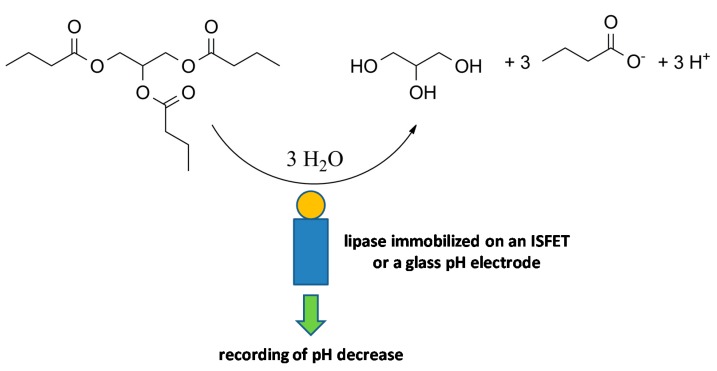
The general principle of a triglyceride assay such as a tributyrin (in the figure) assay by a lipase biosensor recording the change in pH. The tributyrin is hydrolyzed up to glycerol and the butyric acid has an impact on the pH of the solution.

Electrochemical sensors are a platform suitable for the construction of a biosensor or bioassay using lipase as a part recognizing the analyte. Voltammetry like chronoamperometry (amperometry), pulse voltammetry techniques and potentiometry are all suitable for this purpose and their applications are further described. Since esters are hydrolyzed by lipases, which typically generate an alcohol and an organic acid, using a pH electrode with pH meter is a possible way to construct a biosensor. Such a concept of a biosensor was chosen in the study by Kartal and coworkers where lipase from the fungus *Candida rugosa* was immobilized on a glass pH electrode and allowed to hydrolyze tributyrin as a substrate [54]. The released butyric acid caused a decrease of the pH which was recorded by the glass pH electrode. The assay was used for the determination of the pesticide methyl-parathion, which inhibited the lipase and prevented it from medium acidification. The limit of detection for the method was equal to 93 µmol/L and the biosensor exerted a linear range of 65–455 µmol/L for the methyl-parathion. A glass pH electrode with an immobilized lipase by the sol-gel technique was chosen for the assay of olive oil, which also served as a substrate that caused a change of pH after its hydrolysis [55]. A lipase that can be immobilized on the surface of a sensor based on a self-conductor and ion-sensitive field-effect transistor (ISFET) seems to be suitable for this purpose. An ISFET with immobilized lipase from the porcine pancreate used Pojanowska and coworkers for the measurement of various triglycerides [56]. Lipase was entrapped into alginate gel on glass beads coated with keratin and the modified beads were adsorbed on nitrocellulose sheets. They proved the assay for triacetin, tributyrin and triolein, which were measured with a sensitivity 0.022 pH/mmol/L (triacetin), 0.478 pH/mmol/L (tributyrin) and 0.128 pH/mmol/L (triolein). Though the authors did not report the limits of detection, it appears to be around 1 mmol/L for tributyrin when considering the calibration curves. Though the method is not sensitive to determine the traces of triglycerides, it is well suited in an industry such as the food industry for the continuous monitoring of triglyceride content in a processed medium. The principle of a lipase biosensor recording change in pH is depicted in Figure 4.

**Figure 5:**
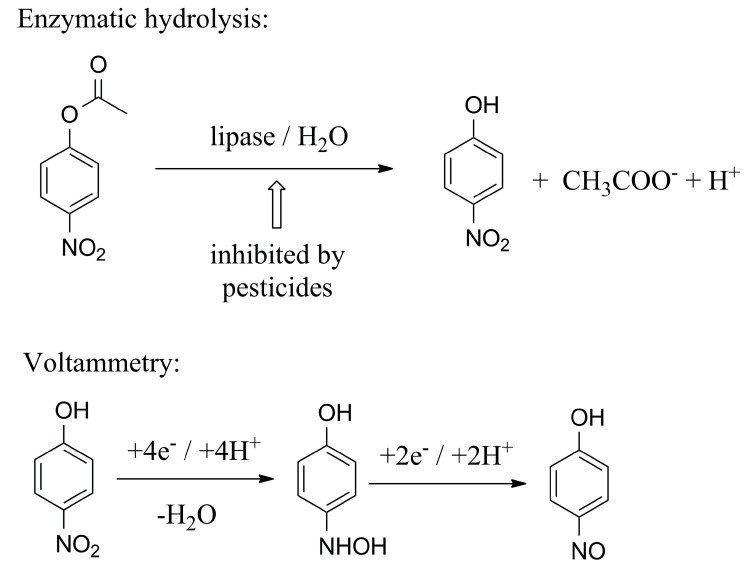
The principle of voltammetry for the determination of lipase inhibitors like malathion, parathion and chlorfenvinphos using *p*-nitrophenyl acetate as the substrate for the enzyme.

Lipase can be also combined with voltammetry and immobilized on the surface of a voltammetric electrode where it initiates the production of an electroactive molecule. This concept was chosen, for instance, by Ma and coworkers in their work on the assay of methyl parathion [57]. The authors immobilized lipase from bacterium *Burkholderia cepacian* on amine-functionalized zeolitic imidazole framework nanoparticles and the modified nanoparticles were captured into chitosan, located on a glassy carbon electrode. The constructed biosensor was suitable for the assay of pesticides containing the *p*-nitrophenyl moiety in their structure. The moiety became hydrolyzed and then electrochemically oxidized in a cyclic voltammetry measurement. The biosensor was tested on methyl parathion, which was determined with a limit of detection equal to 0.28 µmo/L. A similar principle of the assay was chosen by Reddy and coworkers for an assay of the pesticides chlorfenvinphos and malathion in water samples [58]. Compared to the previous study, the pesticides were not substrates for the enzyme but acted as inhibitors. In the assay, the lipase from *Candida rugosa* converted substrate *p*-nitrophenyl acetate to *p*-nitrophenol and acetate. The produced *p*-nitrophenol was electrochemically oxidized and appeared as a peak at 0.024 V versus the standard calomel electrode. The tested pesticides stopped the enzyme reaction and the assay was suitable to prove a value as low as 84.5 µmol/L for chlorfenvinphos and 282 µmol/L for malathion. The linear response to the presence of the analyte was in the range 100–900 µmol/L for both pesticides. The principle of a voltammetry assay where *p*-nitrophenyl acetate served as the substrate and the pesticides served as analytes can be learned from Figure 5. In electrochemical oxidation, *p*-hydoxylaminophenol is the first reaction product, followed by *p*-nitrosophenol. Under some conditions and on some types of electrodes such as working electrodes composed of gold nanoparticles, the oxidation can lead to *p*-aminophenol being the last oxidation product [59].

Impedimetry is another electrochemical technique that can be combined with lipases. Pesticide diazinon was analyzed on this principle by a biosensor with lipase from *Candida rugosa* and porcine pancreas deposited on a gold electrode covered with a self-assembled monolayer and then glutaraldehyde crosslinked [60]. The lipase converted diazinon to diethyl phosphorothioic acid and 2-isopropyl-4-methyl-6-hydroxypyrimidine, which caused a change in the impedance of the medium. The biosensor exerted a limit of detection of 10 nmol/L when fungal lipase was used and 100 nmol/L when the lipase from porcine pancreas was chosen. The survey of the described lipase-based assays is provided as Table 3.

## 5. Optical Lipase Biosensors and Bioassays

Optical biosensors are a direct competitor of the electrochemical one. Both types of assay are relevant in the current analytical praxis and it is not easy to say which one should be preferred because the cost of materials fluctuates and they are also sensitive to other types of interferences. It can be said that one type of sensor platform is better in some conditions while the other should be preferred under other conditions. The absence of expensive noble metal materials and the possibility to estimate the final coloration by a naked eye in some cases are major advantages. The opportunity to use a small camera as a measuring device is another considerable fact in some assays [61,62]. Cloudiness in samples scattering light, color interferents and possible instability in light source intensity due to accumulator discharging or dirt deposition when a portable device is used are the major disadvantages.

*P*-nitrophenyl butyrate as a substrate was selected by Pliego and coworkers for the determination of lipase activity in a stop flow sequential injection analysis system [63]. The principle of the assay was the same as described in the chapter devoted to the standard methods: *p*-nitrophenyl butyrate was hydrolyzed to *p*-nitrophenol and butyric acid, coloration caused by *p*-nitrophenol was measured photometrically at 415 nm. The authors analyzed lipase B from yeast *Candida antarctica* for validation and then yeast *Yarrowia lipolytica* for further experiments and they were able to analyze its presence in the linear range 0.005–1.6 U/mL. The content of lipase in biological samples was also tested in the work of Krieg and Gauglitz who developed a sensor based on reflectometric interference spectroscopy and the total internal reflection fluorescence suitable for the determination of human pancreatic lipase [64]. A flow through the system with a flow cell containing immobilized lipase and antibodies against lipase labeled with DY-652 were used for the assay purpose. In the presence of lipase in a sample, the labeled antibodies were interacting with solved lipase and not with the immobilized one. The competition caused a decrease of fluorescence emitted at 680 nm after illumination by a laser beam with a wavelength of 640 nm. The assay was suitable for determination of lipase in the range 0.068–3.85 mg/L for total internal reflection fluorescence and 0.560–7.87 mg/L for reflectometric interference spectroscopy.

**Figure 6:**
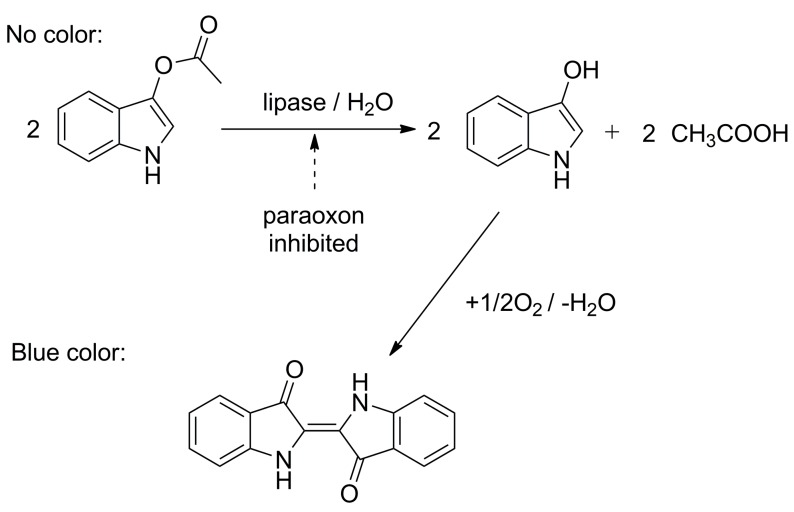
The principle of the colorimetric assay based on indoxyl acetate hydrolysis by lipase which can be inhibited by pesticides like paraoxon.

Optical assays with lipase as a recognition element are also suitable for the measurement of pesticides as shown in the following examples. The colorimetric biosensor with lipase from *Psychrobacter* sp. was used for the measurement of paraoxon ethyl by a camera technique [65]. The assay used the chromogenic substrate indoxyl acetate, providing blue indigo due to conversion by the lipase which was immobilized on the polyvinylidene difluoride membrane. The intensity of the coloration was measured by a camera of a smartphone and the color channels were analyzed. The paraoxon caused the inhibition of the reaction and was analyzed thereof and a limit of detection equal to 37 nmol/L was achieved. The principle of the reaction is shown in Figure 6.

**Figure 7:**
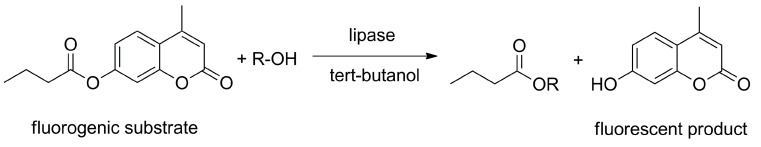
The principle of the fluorometric assay of lipase activity by the transesterification of fluorogenic 4-methyl umbelliferone to fluorescent 4-methylumbelliferone.

A fluorometric analytical method placed on microplates was developed by Zheng and coworkers in order to scale lipase transesterification activity [66]. They performed the assay with two substrates: 4-methyl umbelliferone and methanol in *tert*-butanol. The reaction product, 4-methylumbelliferone, was measured fluorometrically with 330 nm light for excitation and emission at 390 nm. The assay was verified on lipases from fungus *Candida antarctica*, fungus *Mucor miehei*, fungus *Thermomyces lanuginosus*, and bacteria *Pseudomonas cepacia* and *P. fluorescens.* The authors reported full applicability of the assay in lipases characterization. Though the lipases served as analytes itself, the fluorometric assay appears to be fully applicable for other purposes as well. It can be assumed that bioassays based on the fluorometric reaction will appear in the future. The principle of the transesterification reaction is depicted in Figure 7. The survey of lipase-based optical assays is written in Table 4.

## 6. Conclusions

Lipases are a group of enzymes with a huge relevance for bioassays such as biosensors development. They can be easily used in the analysis of various compounds from which pesticides appear to be the most important. The inability to distinguish individual compounds is a major disadvantage in the devices and the assay will point to a certain group of compounds that are able to inhibit the enzyme or to a group of esters that can be hydrolyzed. On the other hand, it is expected that an assay based on lipases will be cheap because both commercially available lipases and devices for assay are well-available on the market and there are no expensive steps in their production or any requirement for a specific material. Lipases are also relevant in human physiology and their activity can be measured for this reason as analytical devices can be used in medical institutions or for home care. Such use will not represent a replacement of standard biochemical tests but may serve as the first screening of a pathology. The simple survey of lipase applications in the analysis by biosensors and bioassays is surveyed in Table 5.

It can be noted that lipases can be applied in a similar situation such as in cholinesterases. Compared to cholinesterases, lipases are less-frequently studied and the number of application for them is lower. It does not, however, mean that lipases are less applicable. Contrarily, lipases are quite cheap, commercially available enzymes that deserve further attention by scholars. The further improvement of lipase-based assays will arise as a consequence of the assays improving by lipase immobilization on nanoparticles or nanostructured membranes, which can improve the analytical parameters and increase the size of the active surface. The practical impact of the recent findings is expected.

## Figures and Tables

**Table 1:** The survey of substrates and products in reactions catalyzed by lipases E.C. 3.1.1.3.

Substrate for Hydrolysis	Reaction Products	Origin of Lipase	References
(*R*,*S*)-ibuprofen + 1-propanol	(*S*)-ibuprofen *n*-propylester + (*R*)-ibuprofen	*Penicillium expansum*	[23]
4-nitrophenyl palmitate + water	4-nitrophenol + palmitate	*Penicillium expansum*	[23]
1,2,3-trihexaicosanoylglycerol + water	1,2-dihexaicosanoylglycerol + hexaicosanoate	*Saccharomyces cerevisiae*	[24]
trimyristin + water	dimyristin + myristate	*Saccharomyces cerevisiae*	[24]
tripalmitin + water	dipalmitin + palmitate	*Saccharomyces cerevisiae*	[24]
tributyrin + water	dibutyrin + butyrate	*Bacillus subtilis, Chromobacterium viscosum*, *Micrococcus* sp., *Rhizomucor miehei*, *R. oryzae*, *Fusarium solani*, wild boar (*Sus scrofa*), humans *Homo sapiens*	[25,26]
α/β-naphthyl stearate + water	α/β-naphthol + stearate	*Micrococcus* sp.	[26]
β-naphthyl butyrate + water	β-naphthol + butyrate	*Micrococcus* sp.	[26]
β-naphthyl laureate + water	β-naphthol + laureate	*Micrococcus* sp.	[26]
4-nitrophenyl esters (laurate, oleate, palmitate, propionate) + water	4-nitrophenol + laurate, oleate, palmitate respective propionate	*Cephaloeia presignis*	[27]
α-naphthyl acetate + water	α-naphthol + acetate	*Cephaloeia presignis*	[27]
methyl esters (acetate, butyrate, palmitate, propionate, stearate) + water	methanol + acetate, butyrate, palmitate, propionate, respective stearate	*Cephaloeia presignis*	[27]

**Table 2:** The inhibitors of lipases E.C. 3.1.1.3.

Inhibitor of Lipase	Origin of Lipase	References
Cd (II+)	*Yarrowia lipolytica*	[32]
Co (II+)	*Mycobacterium tuberculosis*	[33]
Fe (III+)	*Pseudomonas fragi*	[34]
Hg (II+), Ni (II+), Cu (II+), Zn (II+)	*Yarrowia lipolytica*	[35]
alginic acid	wild boar (*Sus scrofa*)	[36]
sodium dodecyl sulfate, *n*-dodecyltrimethylammonium bromide, sorbitan monooleate	*Pseudomonas fragi*	[34]
sodium cholate, sodium lauryl sulfate, *p*-hydroxymeruribenzoate, *N*-bromosuccinimide, acetylacetone, 4-dimethylaminobenzaldehyde	*Rhodotorula mucilaginosa*	[38]
galacturonic acid and pectin	wild boar (*Sus scrofa*)	[39]
1-butanol, 1-propanol, 2-propanol, acetone, acetonitrile, benzyl alcohol, iso-amyl alcohol, iso-butanol, phenylmethylsulfonyl fluoride	*Geobacillus* sp.	[40]
Orlistat (tetrahydrolipstatin)	humans *Homo sapiens*, *Rhizopus oryzae*, whiteleg shrimp *Litopenaeus vannamei*	[41,42,43]

**Table 3:** The overview of lipase-based electrochemical assays.

Origin of Used Lipase	Principle of Lipase Use in the Assay	Analyte	Limit of Detection	References
fungus *Candida rugosa*	lipase was immobilized on a glass pH electrode and converted tributyrin, which caused a decrease of pH; methyl-paraoxon stopped the reaction	methyl-parathion	93 µmol/L	[54]
porcine pancreate	lipase was immobilized on an ISFET and hydrolyzed triglycerides as an analyte, a change in pH was recorded	triacetin, tributyrin and triolein	around 1 mmol/L	[56]
bacterium *Burkholderia cepacia*	lipase was immobilized on zeolitic nanoparticles and then into chitosan on a glassy carbon electrode, pesticides like methyl parathion were hydrolyzed to *p*-nitrophenyl that was electrochemically oxidized in the next step	methyl parathion	0.28 µmo/L	[57]
fungus *Candida rugosa*	lipase converted *p*-nitrophenyl acetate to *p*-nitrophenol and acetic acid, *p*-nitrophenol was oxidized and a current at 0.024 V was recorded, analytes inhibited lipase and stopped the reaction	chlorfenvinphos, malathion	84.5 µmol/L for chlorfenvinphos and 282 µmol/L for malathion	[58]
fungus *Candida rugosa* and porcine pancreas	lipase converted diazinon to diethyl phosphorothioic acid and 2-isopropyl-4-methyl-6-hydroxypyrimidine. which caused a change in the impedance of the medium	diazinon	10 nmol/L (fungal lipase), 100 nmol/L (porcine pancreas lipase)	[60]

**Table 4:** The overview of lipase-based optical assays.

Origin of Used Lipase	Principle of Lipase Use in the Assay	Analyte	Limit of Detection	References
fungus *Candida antarctica* and *Yarrowia lipolytica*	*p*-nitrophenyl butyrate hydrolysis to butyric acid and *p*-nitrophenol, coloration caused by *p*-nitrophenol was measured	lipase itself	0.05 U/mL	[63]
human pancreatic lipase	flows through assay, lipase competed with another immobilized lipase for a fluorescent-dye-labeled antibody, a decrease of fluorescence was measured	lipase itself	0.068 mg/L	[64]
*Psychrobacter* sp.	lipase hydrolyzed indoxyl acetate and blue indigo arose, paraoxon stopped the reaction, the intensity of coloration was measured by camera	paraoxon ethyl	37 nmol/L	[65]
fungus *Candida antarctica*, fungus *Mucor miehei*, fungus *Thermomyces lanuginosus*, and bacteria *Pseudomonas cepacia* and *P. fluorescens*	4-methyl umbelliferone and methanol in tert-butanol were trans-esterified in the presence of lipase, production of 4-methylumbelliferone was measured fluorometrically	lipase itself	n/a	[66]

**Table 5:** The general types of bioassays related to lipases.

Analyte	Role of Lipase	Expected Application
triglycerides, pesticides, various esters	lipase converts the analyte and the reaction is measured	environmental control, agriculture, food industry etc.
pesticide or other toxic compounds	analyte inhibits lipase	environmental control, agriculture, military or police forces etc.
lipase itself	there is measured lipase activity in the sample	health care, providing healthcare outside hospitals, small medical institutions
